# An effective strategy for influenza vaccination of healthcare workers in Australia: experience at a large health service without a mandatory policy

**DOI:** 10.1186/s12879-015-0765-7

**Published:** 2015-02-06

**Authors:** Kristina Heinrich-Morrison, Sue McLellan, Ursula McGinnes, Brendan Carroll, Kerrie Watson, Pauline Bass, Leon J Worth, Allen C Cheng

**Affiliations:** Infection Prevention and Healthcare Epidemiology Unit, Department of Infectious Diseases, Alfred Health, P.O. Box 315, Prahran, VIC 3181 Australia; Public Affairs Unit, Alfred Health, Prahran, Australia; Department of Epidemiology and Preventive Medicine, Monash University, Melbourne, Australia

**Keywords:** Influenza, Vaccination, Healthcare worker

## Abstract

**Background:**

Annual influenza vaccination of healthcare workers (HCWs) is recommended in Australia, but uptake in healthcare facilities has historically been low (approximately 50%). The objective of this study was to develop and implement a dedicated campaign to improve uptake of staff influenza annual vaccination at a large Australian health service.

**Methods:**

A quality improvement program was developed at Alfred Health, a tertiary metropolitan health service spanning 3 campuses. Pre-campaign evaluation was performed by questionnaire in 2013 to plan a multimodal vaccination strategy. Reasons for and against vaccination were captured. A campaign targeting clinical and non-clinical healthcare workers was then implemented between March 31 and July 31 2014. Proportional uptake of influenza vaccination was determined by campus and staff category.

**Results:**

Pre-campaign questionnaire responses were received from 1328/6879 HCWs (response rate 20.4%), of which 76% were vaccinated. Common beliefs held by unvaccinated staff included vaccine ineffectiveness (37.1%), that vaccination makes staff unwell (21.0%), or that vaccination is not required because staff are at low risk for acquiring influenza (20.2%). In 2014, 6009/7480 (80.3%) staff were vaccinated, with significant improvement in uptake across all campuses and amongst nursing, medical and allied health staff categories from 2013 to 2014 (*p* < 0.0001).

**Conclusions:**

A non-mandatory multimodal strategy utilising social marketing and a customised staff database was successful in increasing influenza vaccination uptake by all staff categories. The sustainability of dedicated campaigns must be evaluated.

## Background

Annual influenza vaccination is recommended due to antigenic change in circulating influenza virus stains and the relatively short-lived immunity achieved by immunisation. Influenza vaccination has been shown to be moderately protective against influenza [1]. Health care workers (HCWs) are a target group for influenza vaccination because of their contact with susceptible patients during the course of employment [2,3]. Due to poor reported vaccine coverage, there have been calls for mandatory vaccination policies for staff, including influenza vaccination [4,5], although these have not yet been adopted in Australia.

In 2013, only 56% of HCWs at our health service were knowingly vaccinated against influenza. Recent data from US centres suggests that promotion of vaccination in settings where vaccination is not required (i.e. non-mandatory) can significantly increase uptake [6], and others have demonstrated high vaccine uptake with non-mandatory programs, especially when these effectively engage medical staff [7]. We describe the development and implementation of a successful campaign to improve staff influenza vaccination at our health service in 2014.

## Methods

Alfred Health is a tertiary referral health service in Melbourne, Australia with approximately 7000 staff employed across three campuses. The service has a Staff Immunisation and Exposure Management Unit which provides government-funded influenza vaccination free of charge to staff. Annually, mass vaccination days are held at each campus and are supported by mobile immunisation services. In 2013, the additional resources available for the influenza immunisation program were 1.7 equivalent full-time (EFT) staff for 12 weeks.

### Formative research

To plan and inform the 2014 staff influenza vaccination program, we surveyed staff towards the end of the 2013 program (July 2013) at all campuses regarding their influenza vaccination status and barriers and enablers to influenza vaccination. Electronic (email) invitations containing a link to a web-based survey tool were used to recruit employed staff. The survey contained 10 questions, allowing staff to nominate reasons for vaccination or non-vaccination (see Appendix). Approval to perform the survey was obtained from the Alfred Health Human Research Ethics Committee. Participation was voluntary and anonymity of respondents was preserved.

### Intervention

The 2014 HCW influenza vaccination program was implemented between March 1 and July 31, 2014. The program consisted of the following components:

- *Vaccine availability*
  Immunisation nurses were available throughout the vaccination program on wards and during routine multidisciplinary meetings to offer the influenza vaccination to HCWs. The number of mass influenza vaccination days increased from 3 in 2013 (one each at each campus) to 5 in 2014 (two at The Alfred campus, two at the Caulfield campus and one at the Sandringham campus), with additional days allocated to the 2 larger campuses within our health service. Staff resources available for vaccination outside of the mass vaccination days were comparable to additional resources allocated for the 2013 program, but were focussed earlier in the campaign (2.9 EFT for 8 weeks).
- *Communication*
  Information regarding staff influenza vaccination sessions was provided in weekly electronic communiqués with the support of senior executive and short presentations with a strong public health message were delivered at various hospital-wide meetings. A small campaign sticker was developed for placement on staff identification badges of vaccinated HCWs so that nurse immunisers could quickly identify those staff who had already received influenza vaccine. Posters and screensavers for network computers were locally developed and displayed across all three campuses.
- *Marketing*
  In preparation for the 2014 HCW influenza vaccination program, the Public Affairs Unit at Alfred Health was engaged to formulate a social marketing campaign to improve staff influenza vaccination uptake. Key elements of this campaign included:

- Development of targeted messages to address perceived barriers to influenza vaccination;
- Improved marketing of mass influenza vaccination days, including enhanced communication strategies and provision of incentives for vaccinated staff.

**Figure 1:**
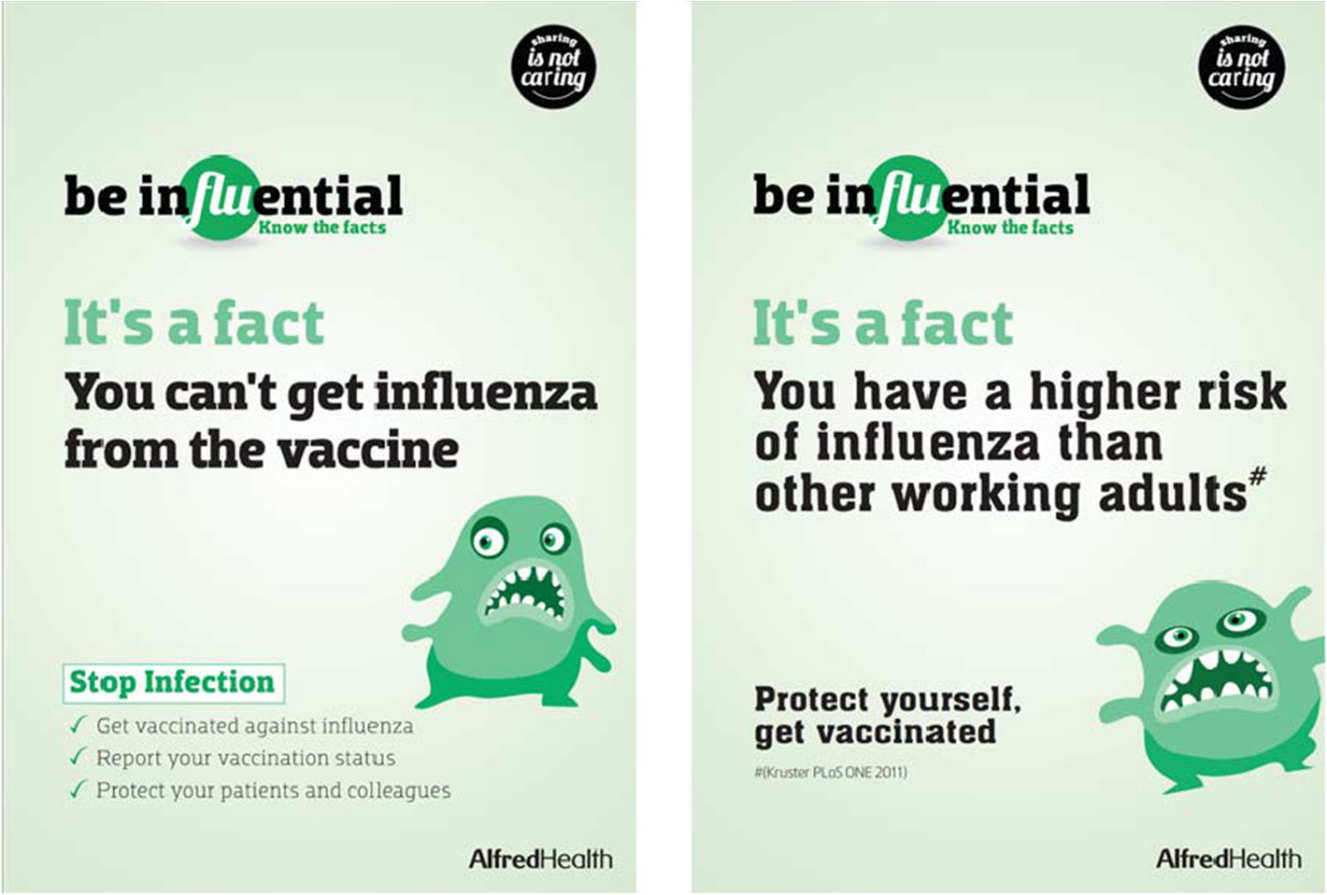
**Social marketing strategy employed for the 2014 staff influenza vaccination campaign.** Examples of poster content, incorporating themes of “sharing is not caring” and “be inFLUential”.

Marketing was based around general framing and specific targeted messages. The general framing was “sharing is not caring” (applied to all infection prevention activities) and “be inFLUential” (to promote the importance of influenza vaccination amongst peers and colleagues). Specific messages were evidence-based, and focused on the increased risk of HCWs acquiring influenza, the small risks of serious complications from influenza vaccination and the risk of severe complications from natural infection. Examples of marketed content are provided in Figure 1.

d) *Database and reporting*
  In 2013, staff influenza vaccination status was captured and housed in a dedicated portion of the staff payroll database and generation of timely progress reports was not feasible. For the 2014 campaign, a new database was developed to record all staff employed during the campaign, including each staff member’s direct line manager. A listing of total staff was obtained from payroll services, and revised to ensure that staff on leave or who were no longer employed, were not evaluated. An employment category (medical, nursing, allied health, laboratory, other staff with clinical contact, staff without clinical contact) was assigned to all employed staff.
  Staff were assessed as vaccinated or declining vaccination using a declaration form [8]. These data were entered into the database after completion of forms by staff. Data entry was continuously performed with any outstanding data entered by the end of each week. On a weekly basis, names of those staff yet to declare their intention for influenza vaccination were extracted and submitted to managers so they could prompt staff to confidentially report to the Staff Immunisation and Exposure Management Unit. Regular reports of vaccination uptake displayed by ward, medical unit and employment category were disseminated electronically to all Alfred Health staff by hospital executive.
e) *Incentives*
  Free coffee was provided to staff who attended the first 3 hours of the mass vaccination days. Door prizes were also offered and the opportunity for any department achieving over 80% compliance with vaccination to go into a draw to win a coffee machine for their department.

### Outcomes

HCWs were defined as those who were permanently, temporarily or casually employed and who had worked at least one shift at one of the three campuses between March and July 2014, in accordance with reporting requirements of the VICNISS state surveillance program [9]. Staff were further subdivided into clinical staff (those who have contact with patients and/or blood or body substances or infectious material) or non-clinical staff (those without patient contact) [10].

At the conclusion of the program, all HCWs were classified as: (i) vaccinated (either by the Staff Immunisation and Exposure Management Unit or elsewhere based on self-report), (ii) declined to be vaccinated, or (iii) not assessed to report status or to be vaccinated. Sub-contractors and those employed by external agencies, unpaid students and staff employed at the co-located research institutes were not considered staff of Alfred Health but were offered vaccination against influenza under arrangements with their employers.

### Analysis

Survey responses were summarised as proportional outcomes. Influenza vaccination rates for 2013 and 2014 were compared using the chi-squared test, with *p* < 0.05 deemed statistically significant. Stata, version 11 (StataCorp, Tx), was used for analysis.

## Results

### Formative research

Responses were received from 1328 of the 6879 employees at Alfred Health, corresponding to a response rate of 20.4%. The majority of respondents were nurses (*n* = 520), with a smaller proportion of allied health staff (*n* = 366), support staff (*n* = 163) and medical staff (*n* = 90). The majority (74%) reported regular clinical contact with patients. Approximately half of staff were employed on a part time basis, including 439 who had a fractional appointment greater than 0.5 EFT and 151 who were employed <0.5 EFT.

**Figure 2:**
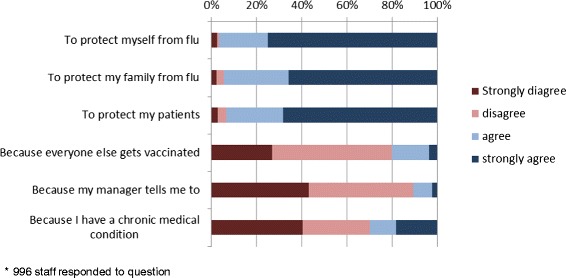
**Vaccinated healthcare workers**: reported reasons for vaccination (2013)*.

1004 staff (75.8% of respondents) self-reported being vaccinated against influenza. Of these, 341 (34.3%) reported being vaccinated in the staff health clinic, 138 (13.9%) reported being vaccinated as part of the mass vaccination days, 425 (42.8%) reported being vaccinated by a mobile vaccination service, and 90 (9.0%) reported being vaccinated outside the hospital (30 at a general practice or pharmacy and 60 at another health service). Reasons for HCWs opting to be vaccinated are summarised in Figure 2, demonstrating the widespread belief that protection of self, family and patients was conferred by vaccination.

**Figure 3:**
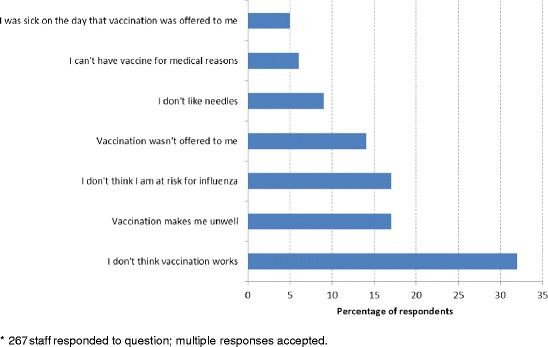
Unvaccinated healthcare workers: reported reasons for choosing not to be vaccinated (2013)*.

321 staff (24.2% of respondents) self-reported not being vaccinated against influenza, of which 62 (21.0%) reported completing a form detailing reasons for declining immunisation. The majority of respondents who were not vaccinated did not complete a form because it was not offered (*n* = 124, 42.0%), they were not aware of the form (*n* = 93, 31.5%) or did not wish to complete the form (*n* = 16, 5.4%). Figure 3 summarises cited reasons for HCWs opting to remain unvaccinated, including beliefs regarding vaccine ineffectiveness (37.1%), that vaccination makes staff unwell (21.0%), and that vaccination is not required because staff are at low risk for acquiring influenza (20.2%).

### Outcomes

In August 2013, 6879 staff were employed at Alfred Health, of which 3866 were known to be vaccinated at Alfred Health with the 2013 trivalent influenza vaccine, 49 staff were reported to be vaccinated elsewhere, 354 declined vaccination and 3504 were not known to be vaccinated.

By August 2014, 6009 of 7480 (80.3%) staff had been vaccinated with the 2014 trivalent influenza vaccine. Of all 7480 staff, 5202 (69.5%) had been vaccinated by the Staff Immunisation Service, 804 (10.7%) had reported being vaccinated elsewhere, 1092 (14.6%) had declined vaccination and 379 (5.1%) had not been assessed for vaccination. There was a significant improvement in vaccination against influenza at all campuses and amongst all staff categories with clinical contact (*p* < 0.0001) from 2013 to 2014 (Table 1). No significant increased uptake was observed among laboratory staff (Table 1).

**Table 1:** **Influenza vaccination uptake at Alfred Health by campus, staff category and clinical department (2013 and 2014)**

	**2013**			**2014**			**P-value** ^**#**^
	**Total staff**	**Vaccinated, n (% of total category)**	**Declined, n (% of total category)**	**Total staff**	**Vaccinated, n (% of total category)**	**Declined, n (% of total category)**	
**Overall**	6879	3866 (56.2)	354 (5.1)	7480	6009 (80.3)	1092 (14.6)	<0.0001
**Campus**							
● The Alfred	4919	3005 (61.1)	310 (6.3)	5647	4579 (81.1)	802 (14.2)	<0.0001
● Caulfield	1312	613 (46.7)	30 (2.3)	1290	987 (76.5)	210 (16.3)	<0.0001
● Sandringham	648	248 (38.3)	14 (2.2)	459	366 (79.7)	73 (15.9)	<0.0001
**Staff category**							
●Nursing	3215	1729 (53.8)	224 (7.0)	3302	2627 (79.6)	527 (16.0)	<0.0001
● Medical	964	656 (68)	25 (2.6)	1224	1066 (87.1)	86 (7.0)	<0.0001
● Allied Health	1387	744 (53.6)	28 (2.0)	1259	1036 (82.3)	155 (12.3)	<0.0001
● Other staff with clinical contact	617	310 (50.2)	51 (8.3)	506	396 (78.3)	73 (14.4)	<0.0001
● Laboratory	134	116 (86.6)	3 (2.2)	222	189 (85.1)	31 (14.0)	0.83
● Staff with no clinical contact	562	311 (50.2)	37 (6.6)	967	695 (71.9)	220 (22.8)	<0.0001
**Clinical department**							
● Emergency	373	184 (49.3)	35 (9.4)	443	335 (75.6)	65 (14.7)	<0.0001
● Intensive Care	408	245 (60.0)	9 (2.2)	509	400 (78.6)	48 (9.4)	<0.0001
● Immunocompromised*	383	226 (59.0)	39 (10.1)	379	331 (87.3)	36 (9.5)	<0.0001
● Other	5153	2900 (56.3)	234 (4.5)	5182	4248 (82.0)	723 (14.0)	<0.0001

*Immunocompromised clinical areas defined as wards nominated for respiratory, lung transplant, haematology (including bone marrow transplantation), medical oncology, and infectious diseases (including HIV) hospital admissions.

^#^Comparison of proportion of healthcare workers vaccinated in 2013 vs. 2014.

## Discussion and conclusions

We describe a successful campaign to improve vaccination against seasonal influenza for HCWs at our health service. Our immunisation program was implemented with only a small increase in resourcing, used to increase vaccine availability as well as developing a social marketing campaign and database support for timely reporting throughout the program.

We identified several enablers and barriers to vaccination in the survey that are similar to those previously described [11,12]. Messages to encourage vaccination should focus on protection against influenza for staff, their families and their patients. Barriers to vaccination, particularly the perception that immunisation does not work, that staff may not be at risk of influenza and adverse effects of immunisation, should be addressed [6]. Although our survey was limited by a low response rate, our findings are similar to those reported elsewhere [13,14]. Our findings also suggested that a significant minority of staff opted for vaccination elsewhere, an important issue to consider where a large proportion of the workforce is employed on a part-time basis.

Despite the limitations, these findings were useful in formulating a promotional strategy to improve influenza vaccine uptake. The application of social marketing principals to healthcare is a useful framework to consider promotional measures to support public health strategies [15]. Our program focused on the “marketing mix” of price (provided free, and addressing perceived barriers), promotion (strategic use of incentives, regular communication and feedback), placement (mass immunisation days supplemented by ward-based services) and product (emphasizing the benefits of vaccination) [16]. A feature of our infection prevention activities is the strong support of senior hospital executive and senior medical staff. This is reflected by high uptake of influenza vaccination amongst medical staff (87.1%) and also other process measures such as compliance with hand hygiene practices (medical staff 81.6%, nursing staff 76.6% compliance in 2014). The staff influenza vaccination campaign forms part of a broader effort to improve patient safety at our health service by preventing infections in staff and patients.

The proportion of staff vaccinated in our health service following this campaign (80.3%) was much higher than we had previously achieved, and higher than other published figures in Australian hospitals [17,18]. In a review of 10 Australian studies, only 3 studies documented vaccination rates of more than 50% [19], with 2 associated with the implementation of active policies or campaigns. Barriers to vaccination included the lack of free vaccine and poor convenience of vaccination services [19]. Reasons for staff non-compliance with our dedicated program were not assessed as part of the current study, but we believe this to be an important consideration in planning of future programs. Our program coincided with a statewide target of 75% vaccine uptake by HCWs being set by the Victorian Department of Health in 2014,[18] and this being introduced as a key performance indicator for Victorian hospitals [20].

Though successful in achieving improved vaccination uptake, our dedicated program did not result in uptake comparable to a recent non-mandatory program implemented at a Japanese centre [7]. This strategy achieved a 97% vaccination uptake, but was implemented at a smaller single-site hospital and included interviewing of non-compliant staff by hospital executive. Our program spanned larger and multiple hospital campuses, and did not involve direct liaison of hospital executive with employees, and this may explain the observed differences in vaccine uptake.

Mandatory vaccination policies [21] are recommended by the Centers for Disease Control and Prevention for hospitals in the United States. While we strongly support the use of influenza vaccination to protect staff and patients, we have previously outlined reasons why we do not believe a mandatory vaccination policy is justified for influenza [22]. These include the moderate effectiveness of the vaccine [23], the lack of data suggesting that nosocomial transmission is a significant problem [24], and the availability of alternative, less restrictive policies to achieve the same goals. Additionally, mandatory influenza vaccination policies present new challenges, including the need for staff redeployment or the wearing of masks [25]. A survey of healthcare workers in two NSW hospitals demonstrated poor support for mandatory policies for influenza vaccination [26], and this has also been voiced more broadly by national stakeholders involved in vaccination policy or program implementation [27].

In summary, we have developed and implemented a successful campaign to improve influenza vaccine uptake at a large Australian healthcare facility. This campaign was informed by a staff survey, and included social marketing, feedback to managers and improving the availability of vaccination. Use of comparable strategies in other centres without mandatory programs would potentially increase vaccine uptake.

## Appendix

**Staff questionnaire: barriers and enablers to influenza vaccination (2013)**

1. **What is your job role?**
  - doctor
  - nurse
  - allied health
  - support services
  - volunteer
  - student
  - other
2. **Which campus do you work at?**
  - Alfred hospital
  - Caulfield hospital
  - Sandringham hospital
  - off-site (e.g. hospital-in-the-home)
3. **Do you have direct contact with patients?**
  - yes
  - no
4. **Do you work full time or part time?**
  - full time
  - part time (≥0.5 EFT)
  - part time (<0.5 EFT)
  - casual or irregularly
5. **Were you vaccinated against the flu this year?**
  - yes
  - no
6. **What are good reasons to be vaccinated?**
  - to protect myself from flu
  - to protect my family from flu
  - to protect my patients
  - because everyone else gets vaccinated
  - because my manager tells me tobecause
  - I have a chronic medical condition
7. **Where were you vaccinated?**
  - at Alfred Health – in the staff immunisation clinic
  - at Alfred health – as part of the flu launch day
  - at Alfred health – when a vaccinator came to my ward or area
  - at a general practice or pharmacy
  - at another hospital or health service
8. **Why didn’t you have the flu vaccine this year?**
  - it wasn’t offered to me
  - it makes me unwell
  - I can’t have it for medical reasons (e.g. allergy)
  - I was sick on the day it was offered to me
  - I don’t think it works
  - I don’t think I am at risk
  - I don’t like needles
9. **Did you sign a form saying why you didn’t want to be vaccinated?**
  - yes
  - no – it wasn’t offered to me
  - no – I didn’t know there was a form for this purpose
  - no – I didn’t want to
10. ** How can we do better at getting staff vaccinated against flu?**

EFT, equivalent full-time
